# PEDOT:PSS as multi-functional composite material for enhanced Li-air-battery air electrodes

**DOI:** 10.1038/srep19962

**Published:** 2016-01-27

**Authors:** Dae Ho Yoon, Seon Hye Yoon, Kwang-Sun Ryu, Yong Joon Park

**Affiliations:** 1 Department of Advanced Materials Engineering, Kyonggi University, 154-42 Gwanggyosan-ro, Yeongtong-gu, Suwon-si, Gyeonggi-Do, 443-760, Korea; 2 Department of Chemistry, University of Ulsan, Daehakro 93, Muger-dong, Namgu, Ulsan, 680-749, Korea

## Abstract

We propose PEDOT:PSS as a multi-functional composite material for an enhanced Li-air-battery air electrode. The PEDOT:PSS layer was coated on the surface of carbon (graphene) using simple method. A electrode containing PEDOT:PSS-coated graphene (PEDOT electrode) could be prepared without binder (such as PVDF) because of high adhesion of PEDOT:PSS. PEDOT electrode presented considerable discharge and charge capacity at all current densities. These results shows that PEDOT:PSS acts as a redox reaction matrix and conducting binder in the air electrode. Moreover, after cycling, the accumulation of reaction products due to side reaction in the electrode was significantly reduced through the use of PEDOT:PSS. This implies that PEDOT:PSS coating layer can suppress the undesirable side reactions between the carbon and electrolyte (and/or Li_2_O_2_), which causes enhanced Li-air cell cyclic performance.

Currently, Li-air batteries are the most impressive next-generation battery systems, because their energy storage is expected to far exceed that achievable with state-of-the-art Li-ion batteries^1^,^2^,^3^,^4^,^5^,^6^,^7^,^8^,^9^,^10^. However, the physical realization of these devices continues to face several major challenges, such as their significant overpotential, low rate capability, and limited cyclic performance^11^,^12^,^13^,^14^,^15^,^16^,^17^,^18^,^19^. In the aprotic Li-air cell, air electrode stores are charged by the reversible formation and dissociation of Li_2_O_2_. One of the serious problems affecting this mechanism is that side reactions (occasionally called parasitic reactions), such as Li_2_CO_3_ formation and electrolyte decomposition, occur simultaneously during this process. Undesirable reaction products such as Li_2_CO_3_ and organic materials (derived from the electrolyte decomposition) are not easily dissociated on charging and accumulate on the air-electrode surface; this results in increased overpotential and limited Li-air cell cycle life^20^,^21^,^22^,^23^,^24^,^25^,^26^. So, side reaction suppression is a key point of research as regards improving the electrochemical performance of Li-air batteries. It is notable that the side reactions are promoted by carbon, a widely used air electrode base-material. The solid reaction products formed on discharging must be stored in a porous conducting matrix. Carbon is an attractive air-electrode matrix material, because it has high conductivity, low weight, and wide surface area^27^,^28^,^29^,^30^. However, carbon reacts with Li_2_O_2_ to form Li_2_CO_3_ on charging at high voltage, and actively promotes electrolyte decomposition during charging and discharging^27^,^28^,^29^,^30^.

Air electrodes prepared without carbon are a possible solution to this problem. Carbon-free electrodes composed of inorganic materials, such as Co_3_O_4_, gold, and TiC, have been designed and tested by several research groups^31^,^32^,^33^. This approach can considerably suppress the side reaction and improve the cyclic performance; however, the capacity of these substances is significantly lower than that of carbon-based electrodes, because of the high weight of inorganic materials.

Our group has proposed carbon surface modification as a new strategy to prevent carbon instability in the air electrode without significant capacity reduction^34^,^35^,^36^. In our previous studies, stable non-conducting polymer materials such as polydopamine and polyimide were introduced as coating materials for carbon surface modification. It was found that stable polymer layers formed on the carbon surface suppress the side reaction significantly, by limiting direct contact with the electrolyte and/or Li_2_O_2_. So, electrodes composed of coated carbon exhibit superior cyclic performance to electrodes containing pristine carbon, and also have higher capacity than carbon-free electrodes. The polymers (polydopamine and polyimide) previously used for air-electrode coating were non-conducting materials; this can cause the polymer layer to essentially become an obstacle to the redox reaction on the air electrode. So, in these cases, the coating layer must be as thin as possible and some rate capability deterioration is inevitable. However, conducting polymers have high electronic conductivity and can be composited simply, in a sufficient concentration for carbon modification; this may constitute an easy air-electrode fabrication process.

In the present study, encouraged by the successful application of the abovementioned surface-coating method, conducting polymer is proposed as an air-electrode composite material. As a conducting polymer, poly(3,4-ethylenedioxythiophene) polystyrene sulfonate (PEDOT:PSS) is adopted in this work. PEDOT:PSS is a very attractive polymer because it is stable, offers high electronic conductivity, and can be easily composited^37^,^38^. Furthermore, it has been confirmed that PEDOT:PSS exhibits redox activity in Li-air cells^39^, which means that it can play a role in a redox reaction matrix, and it can also act as a coating material for side reaction suppression. In addition, PEDOT:PSS can behave as a conducting binder because of its high adhesion. In this work, the PEDOT:PSS is composited with graphene with a weight ratio of 1:1. (Details of the preparation process are given in the ‘Method’ and Fig. 1). The performance of the composite is compared to that of a pristine graphene sample, in terms of suitability for use as an air electrode. A high concentration of PEDOT:PSS is expected to prove multi-functional, acting as a stable graphene coating material, a redox reaction matrix, and a conducting binder.

## Results and Discussion

The surface morphologies of the pristine graphene and Graphene/PEDOT:PSS composite were first characterized using scanning electron microscopy (SEM) and transmission electron microscopy (TEM) images. As shown in Fig. 2a, the SEM image suggests that the pristine graphene is composed of a group of folded graphene sheets. The particles have a wrinkled-paper-like appearance, with sizes of the order of several micrometers. In contrast, the surface of the Graphene/PEDOT:PSS composite appears to be smooth and covered with a coating layer, which is expected of the PEDOT:PSS (Fig. 2c). The sample surface compositions were compared using energy-dispersive X-ray spectroscopy (EDS). As is evident from the right-hand side of Fig. 2b,d, the Graphene/PEDOT:PSS composite surface exhibits higher S (sulfur) content than the pristine graphene surface, which indicates the presence of the PEDOT:PSS layer. Fig. 2 present the TEM images of the pristine graphene and the composite. While the pristine graphene sample surface is clear, the composite has a darker contrast than the pristine graphene particles, which is due to the PEDOT:PSS layer. The coating layer thickness appears to be slightly inhomogeneous; this is because a simple and facile wet process was used instead of an electrochemical coating method that would have provided a sophisticated coating layer. In our previous studies^34^,^35^,^36^, stable non-conducting polymer layers were introduced for the surface modification of carbon materials. These attempts successfully suppressed the undesirable side reactions on the electrode surface; however, the non-conducting characteristic of the polymer seemed to disturb the electron exchange between the electrode and the reaction products during cycling, which may have deteriorated the rate capability of the cell. So, in such cases, the polymer coating layer thickness should be controlled and minimized using an elaborate procedure to reduce the non-conducting effect of the polymer. However, control of the PEDOT:PSS thickness is less critical, because PEDOT:PSS is a conducting material. This characteristic means that a facile, commercially favorable, fabrication procedure can be used for PEDOT:PSS application.

To characterize the electrochemical performance, air electrodes employing pristine graphene or the Graphene/PEDOT:PSS composite were prepared. For convenience, the electrodes employing the Graphene/PEDOT:PSS composite and the pristine graphene are referred to as the “PEDOT electrode” (no binder) and the “pristine electrode” (containing 10 wt.% polyvinylidene fluoride (PVDF) as a binder), respectively. Fig. 3a,b show the initial discharge-charge profiles of the electrodes at current densities of 400, 1000, and 2000 mA·g^−1^ in the 2.35–4.35 V voltage range. All capacities provided in this article are for the total electrode mass (excluding the weight of the Ni mesh used as a current collector). The capacity of the PEDOT electrode is somewhat smaller than that of the pristine electrode, however, considerable discharge and charge capacity is measured at all current densities. As the most of PEDOT electrode surface is composed of a PEDOT:PSS layer because of large amount of PEDOT:PSS (50 wt.%), this clearly shows that the PEDOT:PSS surface can act as a matrix for redox reaction between Li ions and oxygen, and offer sufficient space for storage of the reaction products. Moreover, the considerable charge capacity of the PEDOT electrode indicates that the PEDOT:PSS surface also exhibits OER (oxygen evolution reaction) catalytic activity, as reported previously in ref. 39.

Somewhat smaller capacity of the PEDOT electrode may be attributed to the lower surface area of the PEDOT:PSS-coated graphene compared to that of the pristine graphene. Actually, the PEDOT electrode has a higher content of active material in the electrode than the pristine electrode because PEDOT electrode does not contain binder. This can result in higher capacity of PEDOT electrode than pristine electrode, because the capacity is calculated based on the total electrode mass. However, as shown in Fig. 2a–d, while the surface of the pristine electrode is porous and rough, the PEDOT electrode presents smooth surface, which indicates that the surface area of PEDOT electrode is smaller than that of pristine electrode. So we can expect that the real active area of the PEDOT electrode may lower than that of pristine electrode, which may reduce the capacity of PEDOT electrode.

Although PEDOT:PSS layer acts as active layer, we cannot exclude the possibility that the redox reaction and reaction product formation may be affected by the air-electrode surface material. To check the reaction products, X-ray diffraction (XRD) patterns and Fourier-transform infrared (FTIR) spectra were obtained after the initial fully discharge process. As shown in Fig. 3c, diffraction peaks related to Li_2_O_2_ can be clearly observed not only in the pristine electrode, but also in the PEDOT electrode. However, the PEDOT electrode diffraction patterns exhibit slightly broader peaks compared to those of the pristine electrode, which implies that the Li_2_O_2_ formed on the former has inferior crystallinity to the Li_2_O_2_ formed on the latter. Moreover, the amount or particle size of the Li_2_O_2_ may affect the diffraction peaks. As shown in Fig. S1, the Li_2_O_2_ of the fully discharged PEODT electrode seems to be smaller than that of the pristine electrode. The FTIR spectra of both electrodes also confirm the presence of Li_2_O_2_ after the discharge process, as shown in Fig. 3d. Considering the XRD and FTIR analyses, it is clear that Li_2_O_2_ can be formed on the PEDOT:PSS surface.

As expected, the discharge capacity is significantly decreased as the current density is increased to 2000 mA·g^−1^ (Fig. 3a,b). One noticeable aspect is that the PEDOT electrode still exhibits considerable capacity at high current densities (1000 and 2000 mA·g^−1^). Also, the rate capability of the PEDOT electrode seems to be similar to that of pristine electrode. Generally, the conductivity of PEDOT:PSS (10−10^2 ^S·cm^−1^) is lower than that of graphene (10^2^−10^4 ^S·m^−1^), which may be responsible for the lower rate capability of the air electrode containing PEDOT:PSS. However, interestingly, the surface resistance of the PEDOT electrode is only 5 Ω·sq^−1^, while that of the pristine electrode is 145 Ω·sq^−1^. The lower conductivity (higher resistivity) of the pristine electrode may be attributed to the non-conducting binder (PVDF). The electrode contains not only graphene with high conductivity, but also 10 wt.% of non-conducting PVDF as a binder, which may reduce the electronic conductivity of the electrode surface. In contrast, the PEDOT electrode does not contain a binder, because PEDOT itself has sufficient adhesion to attach the graphene to the current collector (Ni mesh) surface, *i.e.*, PEDOT acts as a conducting binder as well as a coating material. So, the PEDOT electrode, which is completely composed of conducting materials, has higher electronic conductivity than the pristine electrode. The impedance values of the pristine and PEDOT electrodes are similar, as shown in Fig. S2a, which means the PEDOT:PSS layer does not add resistance to the air electrode. Therefore, it is clear that the Graphene/PEDOT:PSS composite can be used as an air electrode with good catalytic activity and considerable rate capability.

Figure 4 compares the cyclic performance of the pristine and PEDOT electrodes at current densities of 400 mA·g^−1^. The cells were cycled with a limited capacity of 1000 mAh·g_electrode_^−1^ to prevent a large depth-of-discharge^40^. The voltage range was 2.0–4.35 V and the upper potential (4.35 V) was maintained until the current density reached 2 mA·g^−1^ during charging, to facilitate reaction-product decomposition. As shown in Fig. 4, the pristine electrode maintains its capacity (1000 mAh·g_electrode_^−1^) for only 45 cycles. In contrast, the PEDOT electrode exhibits significantly superior cyclic performance (over 100 cycles) to that of the pristine electrode. As shown in Fig. S3a, the discharge-charge profiles of both electrodes at the 25^th^ cycle are very similar. As the cycles are increased to 50 (Fig. S3b), the PEDOT electrode still exhibits a stable discharge-charge profile (although the charge over-potential is somewhat increased). However, the pristine electrode exhibits a dramatically reduced capacity (below 400 mAh·g_electrode_^−1^) and a significantly increased over-potential.

The improved cyclic performance of the PEDOT electrode may be associated with the protective effect of the PEDOT:PSS, *i.e.*, the PEDOT:PSS layer on the air-electrode surface protects the carbon, so the undesirable side reactions, such as Li_2_CO_3_ formation due to the reaction of carbon and Li_2_O_2_, and electrolyte decomposition promoted by the carbon surface, are successfully suppressed^34^,^35^,^36^. Impedance analysis of the electrodes after cycling implies this protective PEDOT:PSS effect. As shown in Fig. S2a, the impedance values of the PEDOT and pristine electrodes are similar before the electrochemical test. However, after 50 cycles, the impedance value of the pristine electrode is significantly increased compared with that of the PEDOT electrode (Fig. S2b); this may be the result of undesirable side reactions.

In order to specifically determine the reduction in side reactions caused by the PEDOT:PSS layer, the SEM images and FTIR spectra of the electrodes after 50 cycles (charged state) were observed, as shown in Figs 5 and 6. The cycling conditions were identical to those of Fig. 4. It can be seen in Fig. 5a that the surface of the pristine electrode seems to be covered with a large number of particles and heterogenous layers. As this is a charged state, this implies that a considerable amount of reaction products from undesirable side reactions accumulate on the surface of the pristine electrode. In contrast, the PEDOT:PSS surface has a smooth appearance, as shown in Fig. 5b, very similar to the pre-test surface (Fig. 2c). Although it is possible that some heterogenous layers form on the electrode surface, it is clear that the accumulation of reaction products is significantly reduced through the use of PEDOT:PSS.

The FTIR spectra collected from the electrodes after the 50^th^ cycle (charged state) also confirm the protective effect of the PEDOT:PSS, as shown in Fig. 6a,b. The 400−500, 600−700, 1350−1500, and 1500−1700 cm^–1^ (marked with ♦) may be associated with undesirable reaction products such as CH_3_CO_2_Li and HCO_2_Li (HCO_2_Li has a similar FTIR spectrum to CH_3_CO_2_Li). The FTIR spectra of both electrodes after 50 cycles contain these peaks; however, the peak intensities of the PEDOT electrode spectrum are significantly lower than those of the pristine electrode. This indicates that the accumulated undesirable side-reaction products can be reduced significantly by introduction of the PEDOT:PSS, *via* its protection of the unstable carbon surface.

## Conclusion

In summary, PEDOT:PSS was successfully introduced as a multi-functional composite material in an air electrode. As Fig. 7 illustrates, PEDOT:PSS functions as a matrix for the redox reaction between Li ions and oxygen, and also stores reaction products because of its high conductivity and catalytic activity. Moreover, it can act as a protective layer to suppress the undesirable side reactions between the carbon and electrolyte (and/or Li_2_O_2_) in the electrode, because it has superior stability to carbon. Finally, it can act as a conducting binder, in place of a non-conducting binder such as PVDF, because of its adhesion. Thus, the multi-functional nature of PEDOT:PSS leads to improved Li-air cell cyclic performance. This study may motivate further active research on management of the undesirable aspects of the commonly available carbon, which will help to realize promising high-specific-energy Li-air cells with enhanced electrochemical performance.

## Methods

### Preparation of Graphene/PEDOT:PSS composite solution

**Figure 1:**
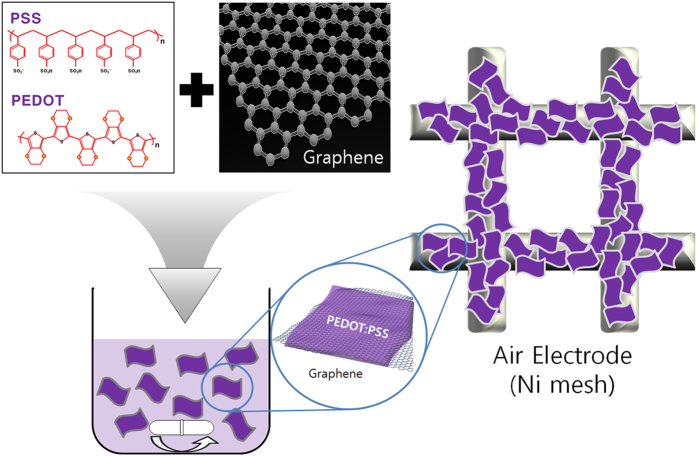
Schematic diagram showing Graphene/PEDOT:PSS composite fabrication process.

**Figure 2:**
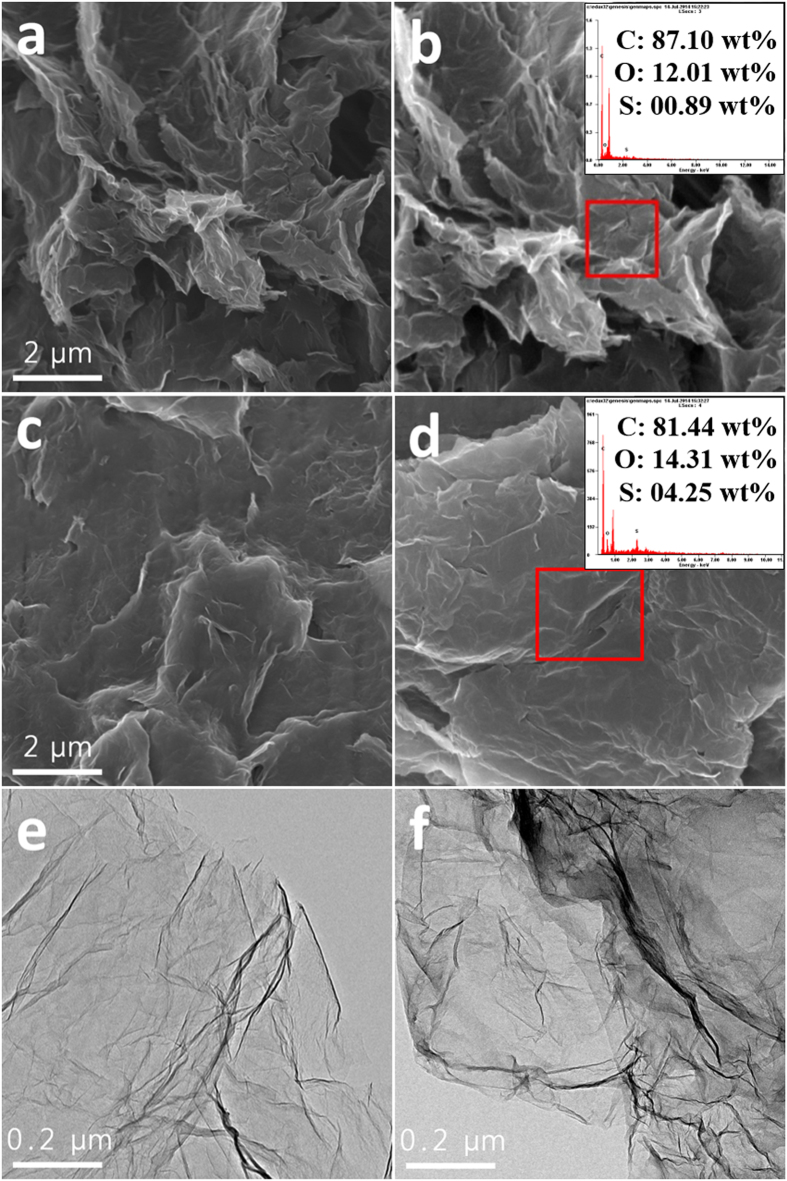
(**a**) SEM image of and (**b**) EDS result for pristine graphene; (**c**) SEM image of and (**d**) EDS result for Graphene/PEDOT:PSS composite; TEM images of (**e**) pristine graphene and (**f**) Graphene/PEDOT:PSS composite.

**Figure 3:**
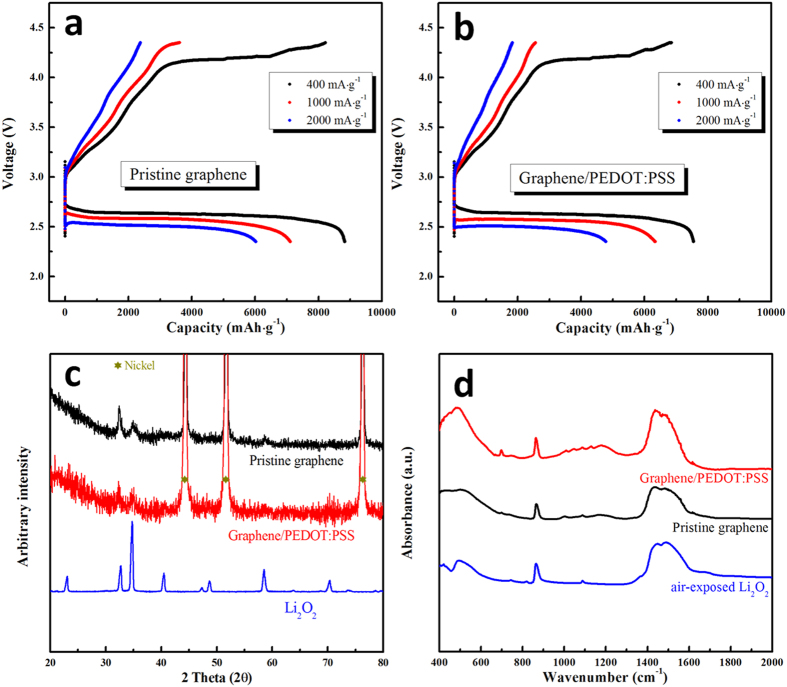
Initial discharge-charge profiles of (a) pristine electrode and (b) PEDOT electrode; (c) XRD patterns and (d) FTIR spectra of electrodes after initial charge process.

**Figure 4:**
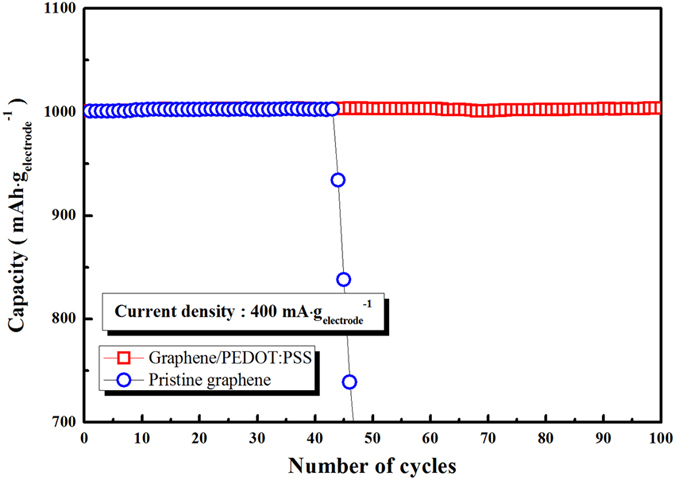
Cyclic performance of electrodes at 400 mA·g^−1^ current density.

**Figure 5:**
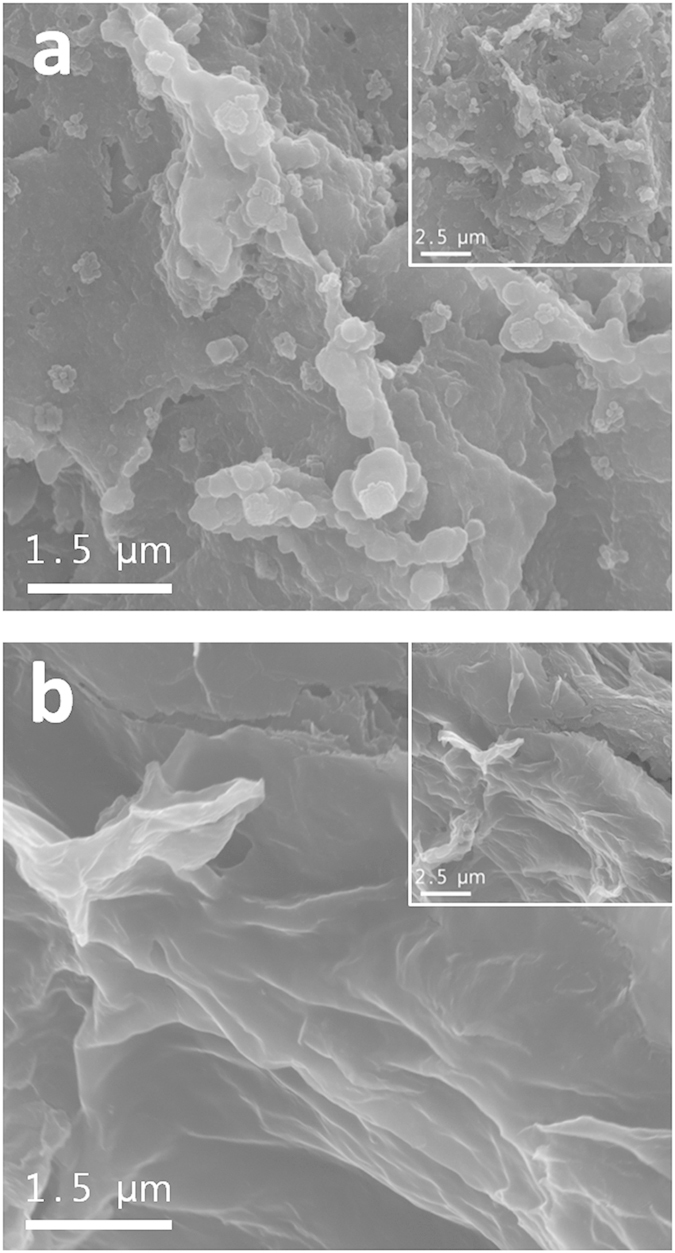
SEM images of (a) pristine electrode and (b) PEDOT electrode after 50^th^ charge.

**Figure 6:**
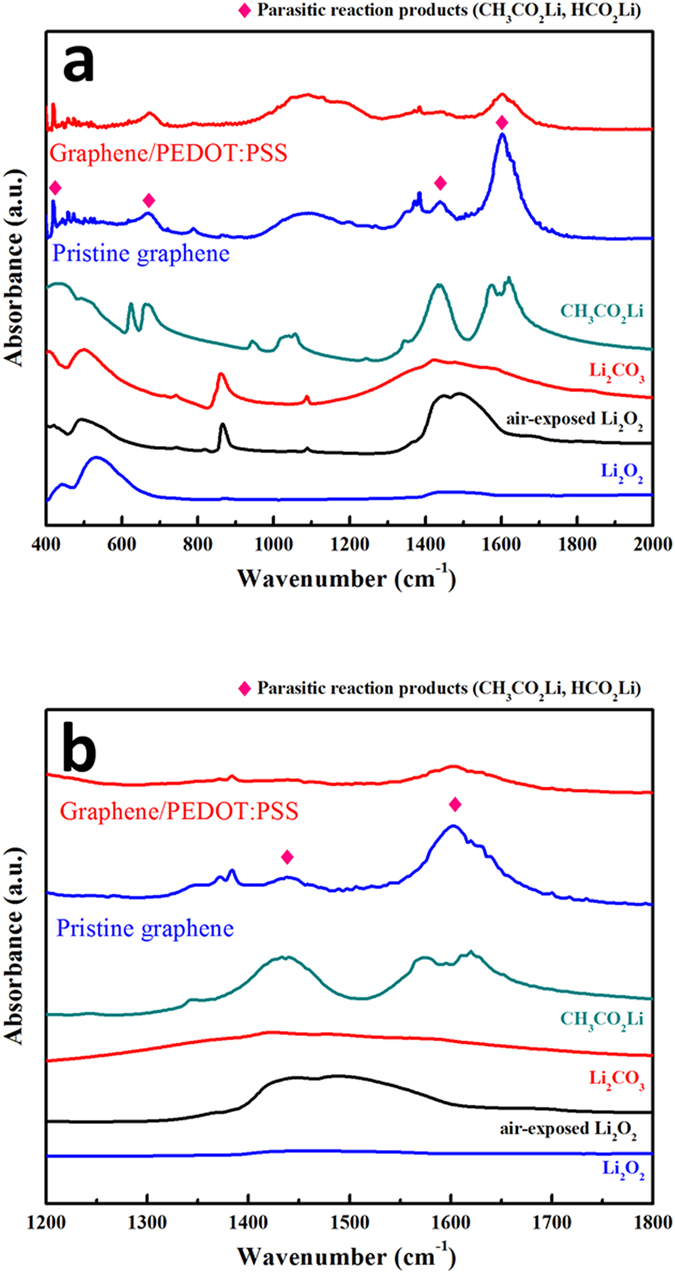
FTIR spectra of pristine and PEDOT electrodes after 50 cycles (charged state). (**a**) 400–2000 cm^−1^ (**b**) 1200–1800  cm^−1^.

**Figure 7:**
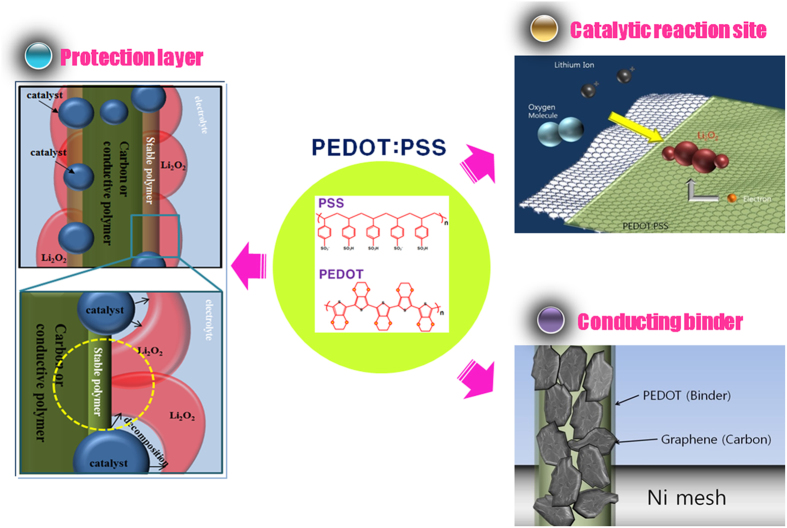
Schematic diagram showing multi-functional effects of PEDOT:PSS. PEDOT:PSS can act as a protection layer suppressing undesirable side reactions, a catalytic reaction site between Li ions and oxygen, and a conducting binder.

To apply the PEDOT:PSS coating to the graphene, pristine graphene was diffused through deionized (D.I.) water *via* an ultrasonic treatment for 30 min. The graphene solution was added to a PEDOT:PSS solution (Aldrich, 1.3 wt.% dispersed in water) at a graphene/PEDOT:PSS weight ratio of 1:1. The final solution was then stirred at room temperature for 2 h in an air atmosphere. Figure 1 is a schematic diagram of the Graphene/PEDOT:PSS composite fabrication process. The microstructures of the pristine and PEDOT:PSS-coated graphene were observed using SEM (AP Tech, TECNAI G2 F30 STwin) and TEM (AP Tech, TECNAI G2 F30 S-Twin). The surface composition of the graphene was also investigated using EDS.

### Preparation of air electrode

Two different preparation methods were used to produce the pristine-graphene and PEDOT:PSS-coated air electrodes. The pristine electrode was prepared by mixing 90 wt.% pristine graphene with 10 wt.% PVDF binder to yield an electrode loading weight of 0.5 mg ± 0.05 mg. The PEDOT:PSS-coated graphene electrode was prepared by heating Graphene/PEDOT:PSS composite solution in order to evaporate the D.I. water to the appropriate viscosity. The evaporated final solution was loaded on a Ni mesh to obtain a loading weight of 0.5 mg ± 0.05 mg.

### Electrochemical testing

The electrochemical performance of the electrodes was examined using a modified Swagelok cell consisting of an air electrode, a metallic Li anode, a Whatman glass filter separator, and an electrolyte made from 1 M LiTFSI in tetraethylene glycol dimethyl ether (TEGDME). The cells were assembled in an Ar-filled glove box and subjected to galvanostatic cycling using a charge-discharge system. All experiments were conducted under an ambient pressure O_2_ atmosphere. The XRD patterns of the electrodes after the initial cycle were obtained using a Rigaku X-ray diffractometer equipped with monochromatized Cu-K_α_ radiation (λ = 1.5406), in order to determine the reaction products. SEM (AP tech TECNAI G2 F30 STwin) was employed to observe the surface morphology of the electrodes after cycling, and FTIR spectra were also collected from the electrodes using a FTIR-4200 (JASCO), in order to ascertain the reaction products accumulated during cycling.

## Additional Information

**How to cite this article**: Yoon, D. H. *et al.* PEDOT:PSS as multi-functional composite material for enhanced Li-air-battery air electrodes. *Sci. Rep.* **6**, 19962; doi: 10.1038/srep19962 (2016).

## Supplementary Material

Supplementary Information
