# Supplementary material for: PEDOT:PSS as multi-functional composite material for enhanced Li-air-battery air electrodes
Source: Sci Rep. 2016 Jan 27;6:19962. doi: 10.1038/srep19962 (PMC4728483; doi:10.1038/srep19962)
Supplement: Supplementary Information [file srep19962-s1.doc]

**Supplementary Information**

**PEDOT:PSS as multi-functional composite material**

**for enhanced Li-air-battery air electrodes**

**Dae Ho Yoon^1^, Seon Hye Yoon^1^, Kwang-Sun Ryu^2^, Yong Joon Park ^1^***


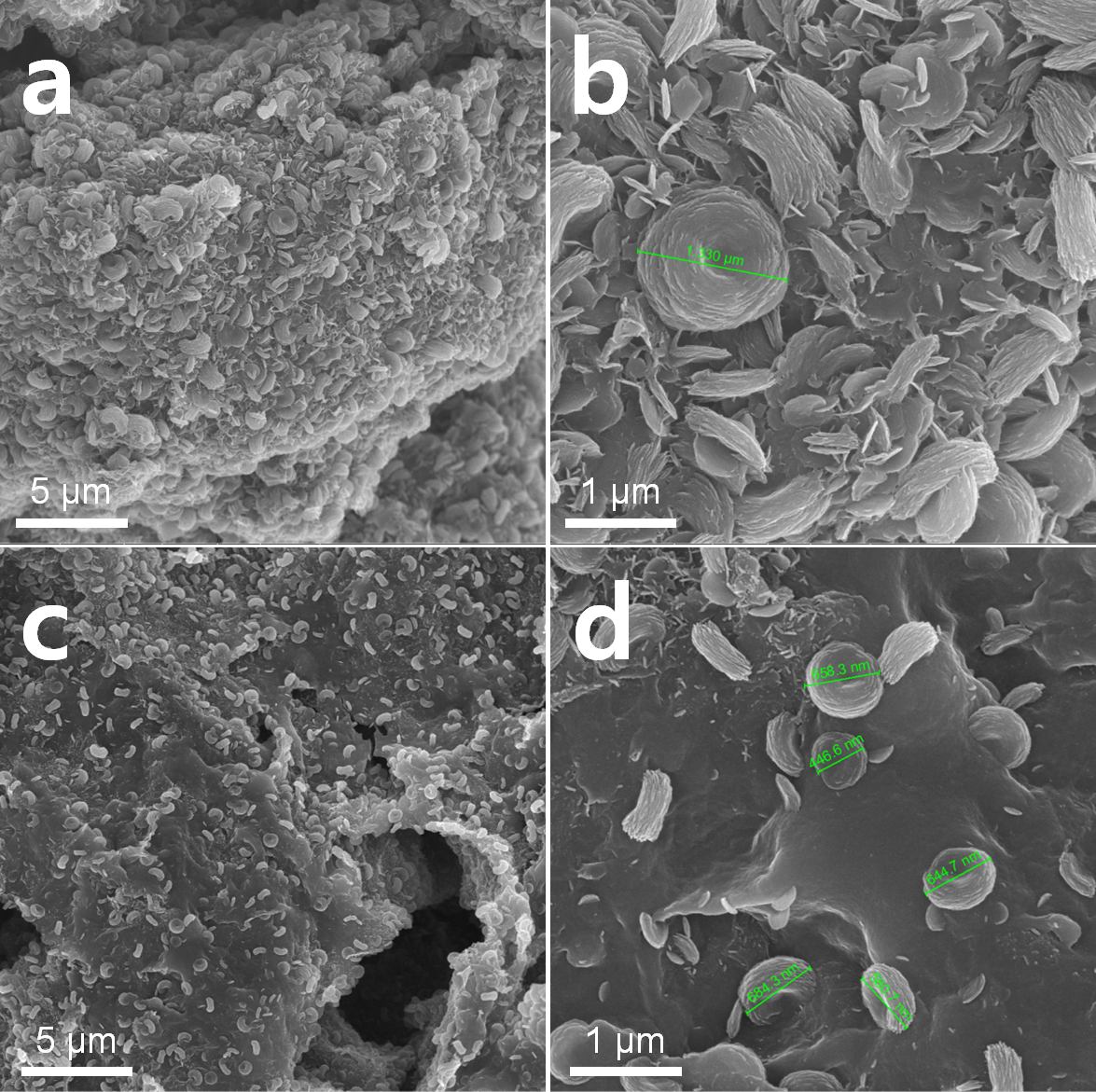


Figure S1. Surface images (SEM) of the electrodes after initial fully discharged. (a, b) pristine electrode; (c, d) PEDOT electrode.


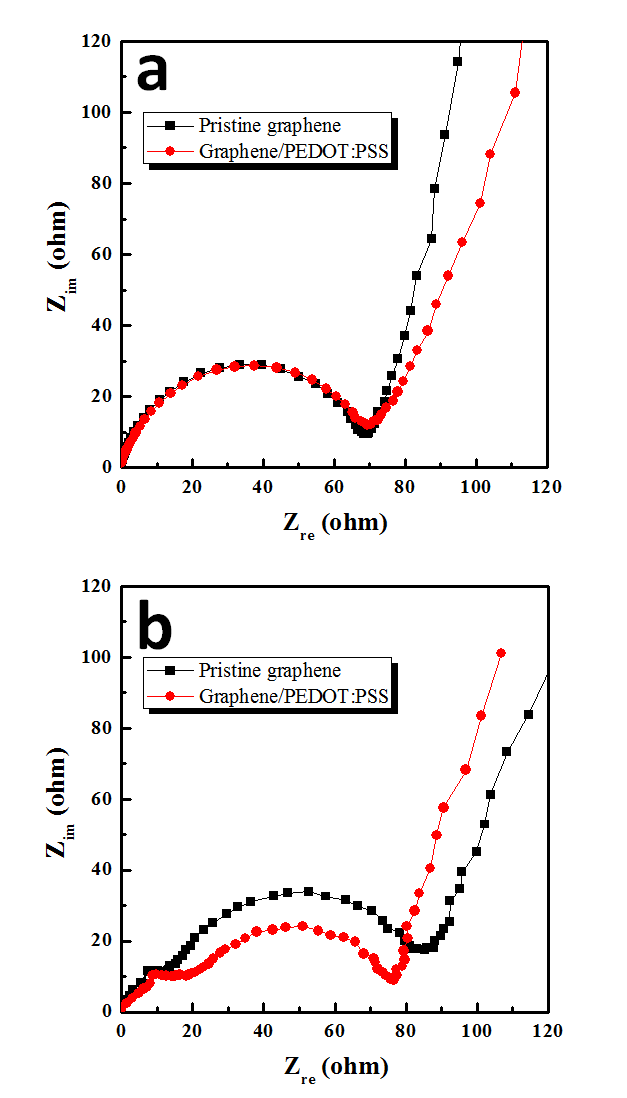


Figure S2. Nyquist plots of pristine and PEDOT electrodes (a) before electrochemical testing and (b) after 50 cycles.


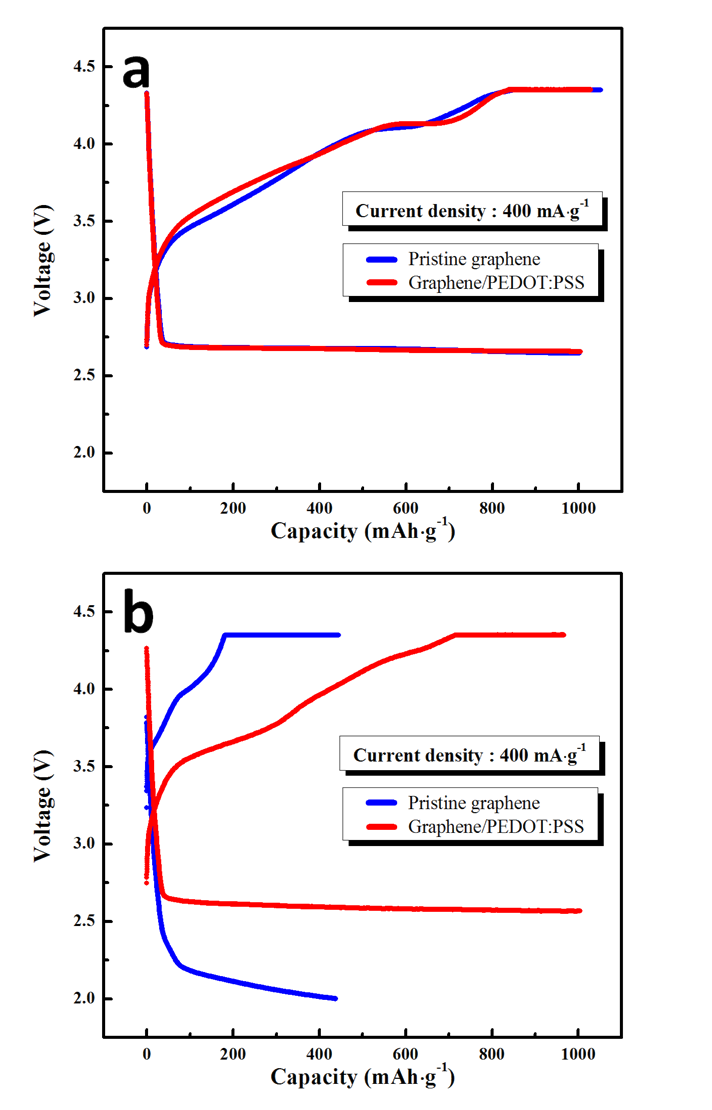


Figure S3. Comparison of the (a) 25th- cycle and (b) 50th-cycle discharge-charge profiles of the electrodes
